# Superconducting quantum circuit of NOR in quantum annealing

**DOI:** 10.1038/s41598-022-20172-0

**Published:** 2022-09-23

**Authors:** Daisuke Saida, Mutsuo Hidaka, Kouhei Miyake, Kentaro Imafuku, Yuki Yamanashi

**Affiliations:** 1 Device Technology Research Institute, National Institute of Advanced Industrial Science and Technology, Central 2, 1-1-1 Umezono, Tsukuba, Ibaraki 305-8568 Japan; 2 Quantum laboratory, Fujitsu research, 1-1 Kamikodanaka, 4-chome, Nakahara-ku, Kawasaki, Kanagawa 211-8588, Japan; 3 School of Engineering Science, Yokohama National University, 79-5 Tokiwadai, Hodogaya, Yokohama, Kanagawa 240-8501, Japan

**Keywords:** Physics, Quantum physics, Qubits

## Abstract

The applicability of quantum annealing to various problems can be improved by expressing the Hamiltonian using a circuit satisfiability problem. We investigate the detailed characteristics of the NOR/NAND functions of a superconducting quantum circuit, which are the basic building blocks to implementing various types of problem Hamiltonians. The circuit is composed of superconducting flux qubits with all-to-all connectivity, where direct magnetic couplers are utilized instead of the variable couplers in the conventional superconducting quantum circuit. This configuration provides efficient scalability because the problem Hamiltonian is implemented using fewer qubits. We present an experiment with a complete logic operation of NOR/NAND, in which the circuit produces results with a high probability of success for arbitrary combinations of inputs. The features of the quantum circuit agree qualitatively with the theory, especially the mechanism for an operation under external flux modulation. Moreover, by calibrating the bias conditions to compensate for the offset flux from the surrounding circuit, the quantum circuit quantitatively agrees with the theory. To achieve true quantum annealing, we discuss the effects of the reduction in electric noise in quantum annealing.

## Introduction

Quantum computation holds the promise of solving some computation problems, that cannot be solved effectively on conventional computers^1,2^. Superconducting quantum circuits are a promising technology for quantum computations. Recently, significant progress is achieved in a gate-type quantum circuit, which has a computing advantage over conventional computers in a specific task^3^. However, error-correctable qubits are required for practical applications. For accurate error corrections, the fabrication of millions to billions of qubits is challenging. An alternative method, that is particularly suitable for solving combinatorial optimization problems, is the use of quantum circuits for quantum annealing (QA). Though a testimony of computational advantage over conventional methods in QA has been still under consideration, QA provides the most practical demonstration of quantum computation in the near term^4–14^. When expressing problems to be solved, QA uses a Hamiltonian with a time-dependent term for initializing the ground state. At the end of the evolution, the ground state represents the lowest-energy configuration for the Hamiltonian, and thus a solution to the optimization problem^15–18^. Conventional QA applies the quantum circuit with unit tile topology like the chimera graph architecture to provide general versatility^6–13^. However, Hamiltonians often include many-body interaction terms and therefore do not fit into the topologies consisted of qubits with two-body interactions. We need to transform the Hamiltonian into a mathematical equivalent so that it can be expressed using two-body interactions. Generally, extra qubits must be introduced when constructing the new Hamiltonian. Specifically, the annealing dynamics can be changed as the spectra structure including the excited state is modified^19,20^. One possible way to construct the problem Hamiltonian without including many-body interactions is to express it as the circuit satisfiability (SAT) problem^21,22^. The ground-state spin logic^23^ allows us to obtain the Hamiltonian when the input and output relationships are expressed by Boolean logic gates (cf : NOR gate)^24–26^. In the superconducting quantum circuit embedded in this Hamiltonian, qubit states play a role of inputs and outputs. We have proposed QA with a native implementation of the problem Hamiltonian for a superconducting quantum circuit composed of flux qubits with all-to-all connectivity, where direct magnetic couplers are utilized instead of variable couplers^24^. The problem Hamiltonian, which has a set of ground states consistent with a given truth table, is implemented for the circuit with no redundant qubits. This direct implementation of the original Hamiltonian is essential for obtaining solutions with high accuracy because the original energy relationship in the Hamiltonian is preserved^24^. Using this unique method, we have demonstrated QA in the Hamiltonian of logic gates and a multiplier^24,26^. This allows us to obtain both a highly accurate solution and expandability of scaling. In this study, we present the operation of a basic logic gate for QA with high accuracy. The mechanism can be explained quantitatively by theory, indicating reliable controllability. Additionally, we also focus on the effects of a reduction of electric noise for true QA. Toward an implementation of large scale of the Hamiltonian expressed by the SAT problem, characteristics of connection qubits are demonstrated.

## Results

### Features of NOR and NAND operation

**Figure 1:**
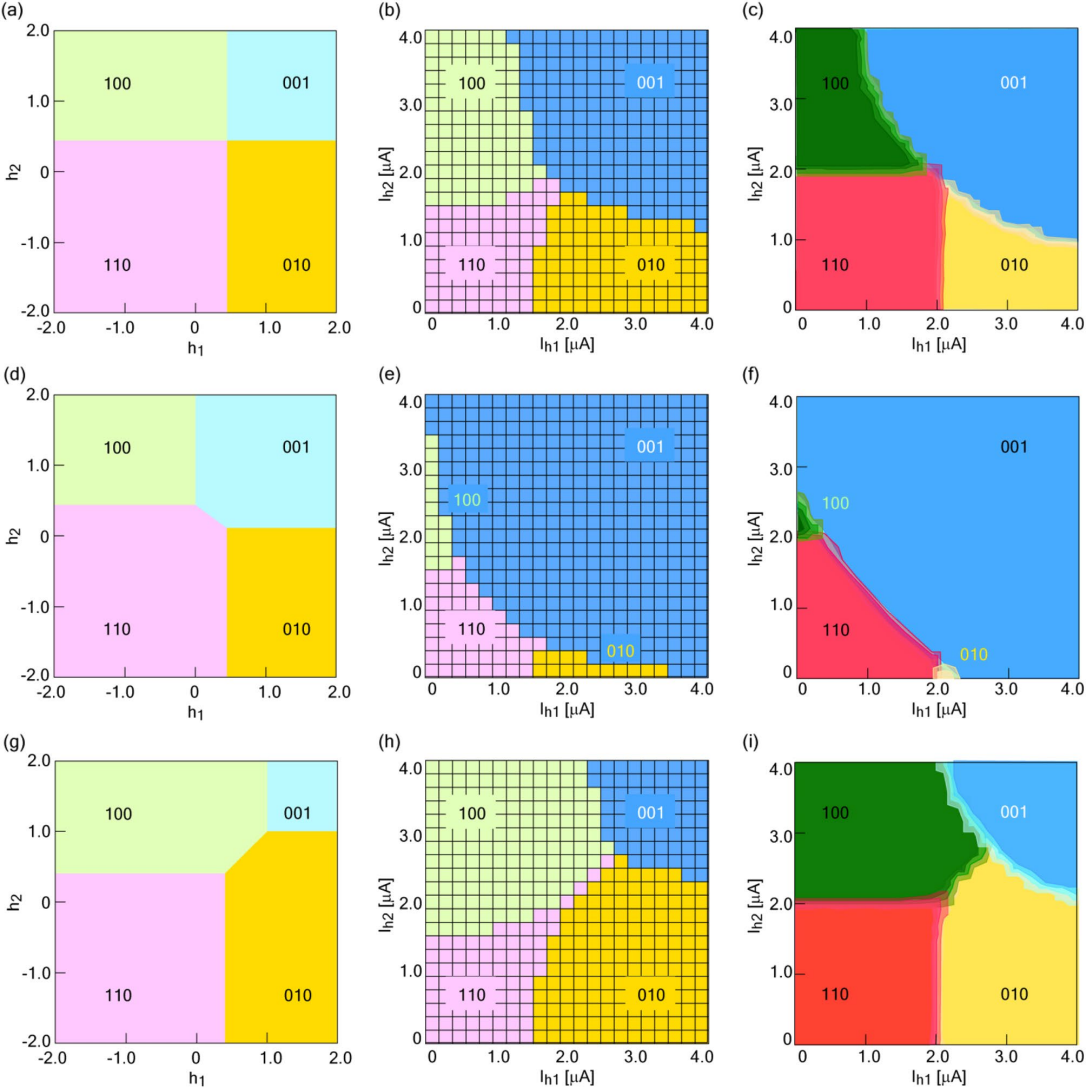
| Trends in state diagrams of NOR operation. State diagrams for (**a-c**) *I*_h3_ = 2.0 µA, (**d-f**) *I*_h3_ = 1.0 µA, (**g-i**) *I*_h3_ = 3.0 µA: (**a**, **d**, **f**) theory, (**b**, **e**, **h**) JSIM analysis without a thermal noise current, and (**c**, **f**, **i**) a 10 mK experiment.

NOR is known to be a versatile computing unit. In our method, the Hamiltonian is designed to minimize energy for logic components of NOR. The superconducting quantum circuit is constructed by directly implementing the Hamiltonian shown in Supplementary Fig. 1(a). Two kinds of samples are prepared consisting of three qubits, corresponding to *A* and *B* for inputs and *R* for the logic result, with critical currents (*I*_c_) of 6.25 µA (NOR1) and 3.75 µA (NOR2). The sample configuration is described in the “Methods” section. The logic components of NOR, corresponding to the four combinations of (*A*, *B*, *R*) with the minimum energy, appear at a degeneracy point after QA. Theoretically, the degeneracy point is expressed as.

$$I_{h2} = \frac{{M_{23} }}{{M_{31} }} \cdot I_{h1}$$,

1 $$I_{h3} = \frac{{M_{1} }}{{M_{3} }} \cdot \frac{{M_{23} }}{{M_{12} }} \cdot I_{h1}$$

where *I*_h*i*_ (*i* = 1–3) is the external bias of qubit *i* (corresponding to labels of *A*, *B,* and *R*), *M*_*i*_ (*i* = 1–3) is the mutual inductance between qubit *i* and the external bias line, and *M*_*ij*_ (*i* = 1–3, *j* = 1–3) is the mutual inductance between qubits *i* and *j*. The process for deriving Eq. (1) is described in the Supplementary Methods. The inductances of the qubits and the mutual inductances between them are extracted from the circuit layout (see Methods). The theoretical degeneracy points of NOR1 and NOR2 are estimated as (*I*_h1_, *I*_h2_, *I*_h3_) = (1.4, 1.4, 3.1) and (1.3, 1.3, 2.8) [µA], respectively. Figure 1a-c respectively shows state diagrams obtained from theory, from simulation using a Josephson integrated circuit simulator (JSIM)^27^ with *I*_h3_ = 2.0 µA, and from an experiment with *I*_h3_ = 2.0 µA carried out at 10 mK. Detailed methods of the JSIM and the experiment are presented in the Methods section and in the “Experimental configuration” section of the Supplementary Methods, respectively. In the JSIM analysis, a thermal noise current is neglected in order to emphasize the trend of the boundary condition in each logic component. A degeneracy point, where every logic component in NOR appears, is found around a current condition of (*I*_h1_, *I*_h2_, *I*_h3_) = (1.8, 1.8, 2.0) [µA] both in experiments and in JSIM analysis at NOR1. Supplementary Fig. 3 shows the frequency distribution of logic components in experiments carried out at the degeneracy point. Logic components corresponding to the minimum energy of the Hamiltonian are selectively generated. In the state diagram, the boundary along the diagonal direction is found, which we call “ladder” for the sake of convenience. The ladder rising diagonally in the left direction is generated when *I*_h3_ decreases from the degeneracy point (Fig. 1d-f). On the other hand, the ladder rising diagonally in the right direction is generated when *I*_h3_ increases from the degeneracy point (Fig. 1g-i). These trends agree qualitatively with theory, JSIM analysis, and experiments. At the experimentally obtained degeneracy point, the logic components of NOR randomly occur (see Supplementary Fig. 4 and the “Detailed characteristics of the NOR operation” section of the Supplementary Note). Note that we can produce a desirable logic component by applying an appropriate offset current (α) against the degeneracy point. For example, the logic component of (*A*, *B*) = (0, 1) can be considered by applying an external flux bias of (*I*_h1_′, *I*_h2_′) = (*I*_h1 _— α, *I*_h2_ + α). This corresponds to adopting α along a diagonal direction from the degeneracy point. By applying an appropriate value of α, NOR logic can be reproduced with high accuracy (see Supplementary Fig. 5). We emphasize that a flux injection to one of the qubits by adopting α in the initial condition restricts the state of the other qubit because the qubits interact with each other to minimize the energy after QA. Moreover, this quantum circuit behaves as NAND when *I*_h_ is supplied with a negative sign. In the state diagram of NAND, the absolute value of the degeneracy point is almost the same as that of NOR. The boundary of each logic component is modulated by *I*_h3,_ as it similarly is in NOR (see Supplementary Fig. 6 and the “NAND operation” section of the Supplementary Note). Each logic component of NAND is reproduced with a probability of success up to 100% by adopting an appropriate value of α (see Supplementary Fig. 7). QA in NOR1 shows a high probability of success in NOR and NAND operation, but its degeneracy point is different among theory, JSIM analysis, and experiments.

### Gray zone evaluation

**Figure 2:**
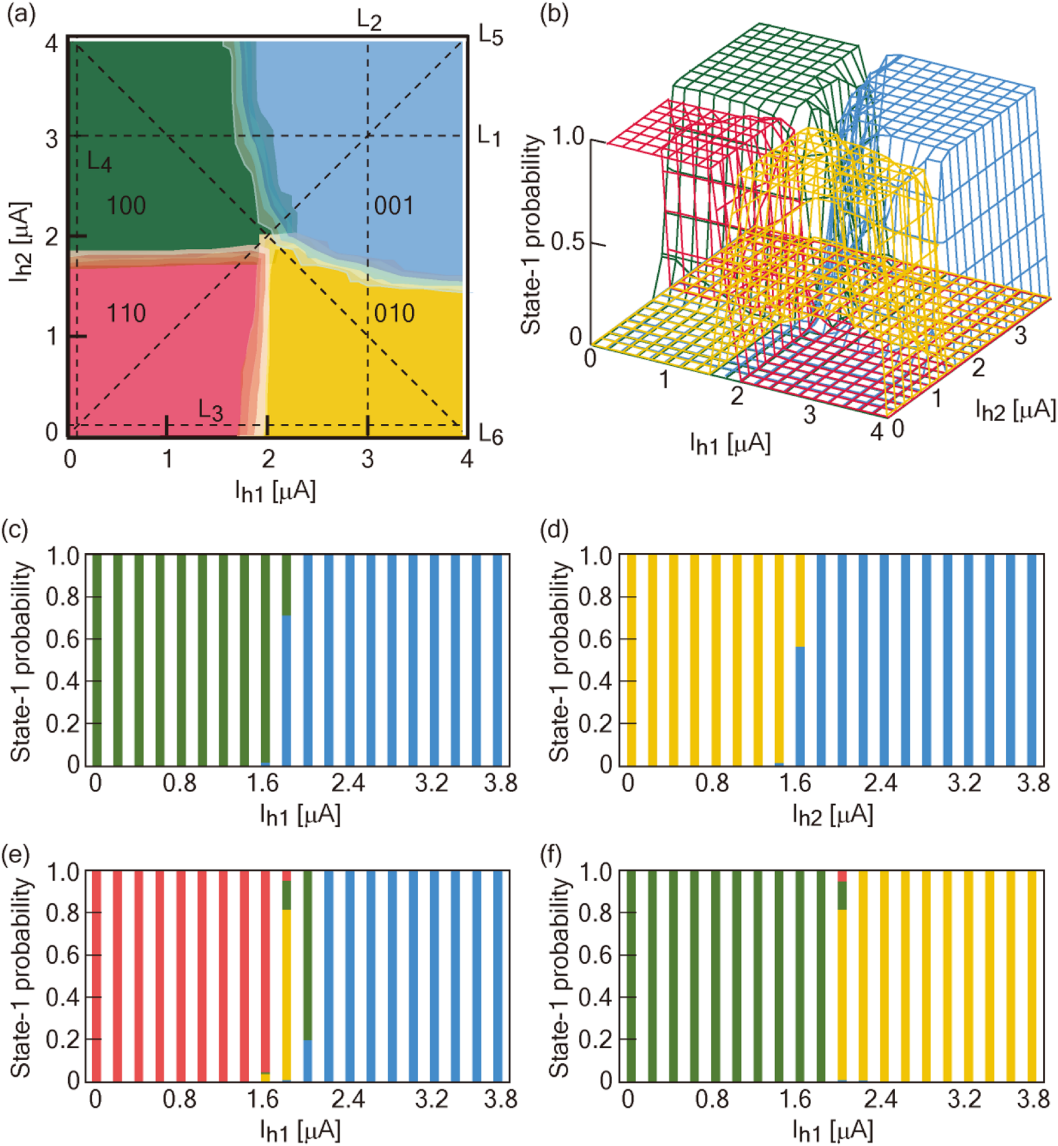
| Analysis of state diagrams in NOR operation. (**a**) Two and (**b**) three-dimensional state diagrams of NOR2 in a 10 mK experiment at *I*_h3_ = 2.8 µA. In order to analyze the boundary width between two logic regions (defined as a gray zone), line profiles of L_1_-L_6_, depicted in (**a**), are evaluated. Line profiles of **(c)** L_1_, (**d**) L_2_, (**e**) L_5_, and (**f**) L_6_.

**Figure 3:**
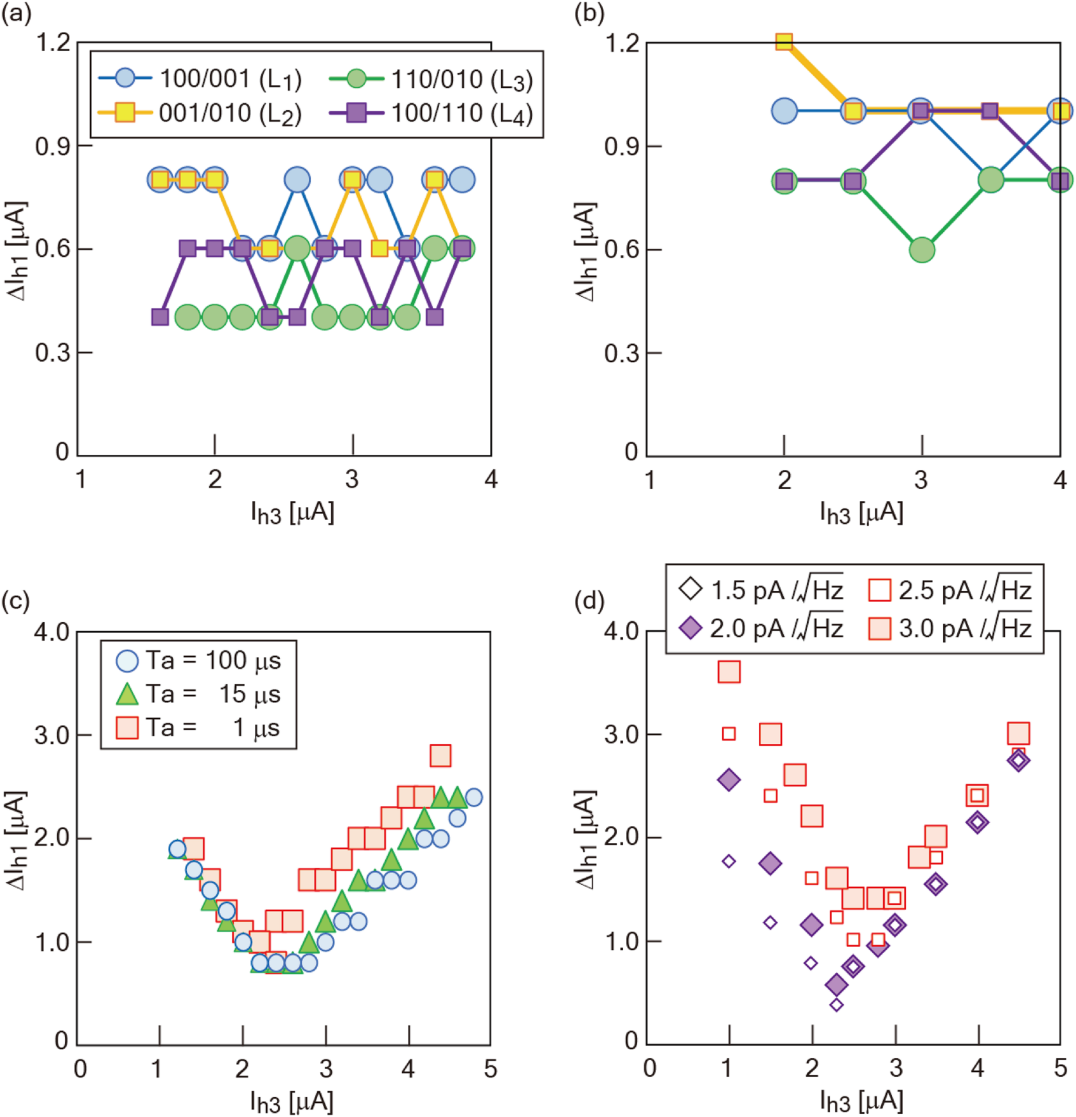
| Gray zone analysis. Type I gray zone analysis in (**a**) 10 mK experiment and (**b**) JSIM analysis. The inset of (**a**) represents the theoretical analysis. In the JSIM analysis of (**b**), a thermal noise current of 3.0 pA/√Hz is adopted. Type II gray zone analysis in (**c**) 10 mK experiment and (**d**) JSIM analysis. NOR2 is utilized.

In NOR2, the barrier height in the energy potential of each qubit is reduced compared with that in NOR1 due to the reduction of *I*_c_. Supplementary Fig. 8 shows the frequency distribution of each logic component with a current condition of (*I*_h1_, *I*_h2_) = (1.6, 1.6) [µA] and a modulation of *I*_h3_ between 0 and 9 µA. Around an *I*_h3_ of 2.8 µA, all candidate logic components in NOR occur. Figure 2a, b show state diagrams with 2D and 3D images at an *I*_h3_ of 2.8 µA. The experimental degeneracy point is close to the theoretical one. Note that the boundary of each logic component drastically changes around this point. The experimental degeneracy point of NOR2 is (*I*_h1_, *I*_h2_, *I*_h3_) = (1.6, 1.6, 2.8) [µA]. For the sake of convenience, we define the transient width between two different logic regions as a “gray zone.” Two types of gray zones exist: Type I is generated between neighboring regions, such as “100”- “001” and “001”- “010”, and Type II occurs in the same diagonal direction as the ladder. Theoretically, the width of the ladder monotonically decreases with external bias *I*_h3_ before the degeneracy point. Later, it monotonically increases with *I*_h3_. Types I and II can be evaluated from four kinds of line profiles (L_1_-L_4_) and the two profiles L_5_ and L_6_, respectively (Fig. 2c-f). In L_5_ and L_6_, four logic components of NOR are identified. Type I gray zones depend on the annealing time (*T*_a_) (see Supplementary Fig. 9 and the “Feature of the Type I gray zone” section of the Supplementary Note). As *T*_a_ decreases, the spread of the gray zone becomes wider. With longer *T*_a_, the effect of noise can be time-averaged. This contributes to the reduction of the gray zone, resulting in the use of the quantum annealing effect. These gray zones are clarified in the case of JSIM analysis with the thermal noise current (see Supplementary Fig. 10). Figure 3a, b show the Type I gray zones evaluated in experiments and in JSIM analysis, respectively. The minimum width of the gray zone differs between experiments and JSIM analysis. The impact of flux generated by surrounding circuits appears differently between JSIM and experiments, which results in a difference in the minimum width of the gray zone. However, the consideration of the equidistant current step in the evaluation of the gray zone contributes to suppressing the effect of minor logic component generation. Gray zones between “100” and “001” and between “001” and “010” tend to be large. These trends correspond to the fact that a boundary position is likely to change due to *I*_h3_ in Fig. 1, indicating an ease in changing the energy state. On the other hand, the values are small in cases of boundaries between “110” and “010” and between “100” and “110”. These trends correspond to the fact that the values of *I*_h1_ and *I*_h2_ do not change with modulation of *I*_h3_ in Fig. 1, indicating difficulty in changing the energy state. These relationships are also confirmed regardless of the value of *T*_a_ (see Supplementary Fig. 9). Figure 3c, d show a Type II gray zone with two trends in experiments and JSIM analysis, respectively. The first is a monotonical response against the absolute value of *I*_h3_ starting from the degeneracy point. This trend agrees with the prediction of the theory. The second is the gray zone spreading slightly wider with the decrease of *I*_h3_ before the degeneracy point than with the increase of *I*_h3_ after the degeneracy point. These trends correspond to the result shown in Fig. 1, where occupation of the “001” region modulates widely with a decrease of *I*_h3_ compared with the case of an increase of *I*_h3_. JSIM analysis also reproduces the same trends seen in experiments. Note that trends change for a thermal noise current above 2.5 pA/√Hz in JSIM analysis. Under 2.0 pA/√Hz, trapping to the local minimum state occurs (see the “Gray zone analysis in JSIM” section of the Supplementary Methods). The logic in NOR and NAND can be realized with high accuracy by tuning the current condition with values of α above 1 µA along a diagonal direction from the degeneracy point, contributing to the avoidance of the gray zone.

## Discussion

Here, we discuss why the theoretical and experimental degeneracy points differ. Degeneracy points agree between theory and JSIM analysis when the thermal noise current is applied. This relates to an escape from trapping to a local minimum energy state in JSIM analysis. We focus on the difference between the experimental results and the JSIM analysis. Here, an offset magnetic flux by a surrounding circuit affects the state transition of a qubit in experiments. We can identify the impact of the offset flux from the trend in the state-1 probability (see Supplementary Fig. 11 and the “Calibration of the degeneracy point in experiment” section of the Supplementary Note). The calibrated degeneracy points are (*I*_h1_, *I*_h2_, *I*_h3_) = (1.6, 1.7, 3.0) [µA] in NOR1 and (1.6, 1.6, 2.9) [µA] in NOR2. Since the smallest half-width of the gray zone is about 0.2 µA, the degeneracy point is almost consistent between theory and experiments. Qubits consisting of NOR1 and NOR2 are designed as the value of a dimensionless factor β_L_ = 2π*LI*_c_/Φ_0_ (Φ_0_ is the flux quantum) of 4.2 and 2.5, respectively, which is designed to construct an energy potential without a local minimum state. As a result, by considering the calibration of the offset flux, the experimental degeneracy point is quantitatively close to the theoretical one. Since the experimental degeneracy point is predictable from consideration of the theoretical one and the offset flux, both high accuracy and controllability are possible with the combination of this superconducting quantum circuit of NOR.

In order to achieve true QA, the suppression of noise effects, including thermal noise, magnetic flux noise from the environment, and electric noise, is vital. The impact of thermal noise is suppressed for experiments carried out at 10 mK. Our experimental setup supports the evaluation of state transitions in the Josephson junction (JJ) with a switching current of 0.28 µA, which is small compared with *I*_c_ in NOR1 and NOR2. The primary origin of noise that affects the Type I gray zone is the noise floor from the dc power supply used for the current source. The dc power supply consisting of a noise floor of order fA/√Hz is used to suppress the electric noise from instruments. Supplying the external bias current *I*_h_ to the circuit of the qubit contributes to eliminating the negligibly small electric noise compared to the thermal energy at 10 mK. Supplementary Fig. 12 represents the expanding width of the gray zone up to 1 µA when the experiment is performed using a dc power supply with a three-orders-of-magnitude higher noise floor compared with the one used in Fig. 1 (see the “Suppression of electric noise toward true quantum annealing” section of the Supplementary Note). In this case, the state transition does not show a dependence on annealing time (see Supplementary Fig. 11(f)). By responding to eliminate the undesirable effect of the electric noise of the instrument, the width of the gray zone is reduced to around 0.4 µA in Type I for the case of the evaluation carried out with the current step of 0.2 µA. Accordingly, QA contributes to the search for a global minimum state in the Hamiltonian, resulting in high accuracy.

It is known that optimization problems that can be effectively handled by QA can be formulated as circuit SAT problems^21,22^. The large scale of the Hamiltonian can be treated by expanding the versatile logic elements of NOR and NAND with a connection qubit. Note that a carry transfer without any error is possible by considering the inductance of the connection qubit (see Supplementary Fig. 13 and the “Expandability of the circuit” section of the Supplementary Note). This means that a superconducting quantum circuit with a wide range of applications will be possible with high expandability in QA.

We have fabricated the superconducting quantum circuit of NOR with a complete logic operation, where the circuit produces arbitrary combinations of inputs with a high probability of success in QA. By selecting an appropriate inductance value, the connection qubit allows us to combine qubits belonging to different unit lattices. In principle, the large scale of the Hamiltonian can be treated by expanding the versatile logic gates with connection qubits. With offset flux calibration, operations in experiments quantitatively agree with the theory. These results contribute to extending the use of quantum computing by providing highly accurate computation for solving Hamiltonians of circuit SAT problems, which are widely applicable to real-world problems.

## Methods

### Superconducting flux qubit

The qubits used in this experiment are superconducting compound JJ rf-SQUID flux qubits, which is a similar configuration to that described by Harris et al^12,13^. We fabricate the superconducting quantum circuit using a process that creates four Nb layers and a JJ with a critical current density of 1 µA/µm^2^. When a flux of Φ_0_ is applied to an inserted small loop with two JJs (the switching current of single JJ is defined as *I*_c_), the rf-SQUID takes two bistable states with persistent current flowing clockwise or counterclockwise through the main loop shown in Supplementary Fig. 1(b). These two states correspond to logical 1 and 0 states in the qubit. Measurement details are described in the “Experimental configuration” section of the Supplementary Methods.

### Design of the NOR

The superconducting quantum circuit of NOR is composed of three qubits with all-to-all connectivity, utilizing direct magnetic couplers instead of variable couplers in the conventional QA circuit^6–13^. Inductances (*L*) and mutual inductances (*M*) are extracted from the layout of the superconducting quantum circuit using InductEX^28^ (see Supplementary Table 1). In the qubit, a bistable energy state can be achieved by coordinating the value of β_L_. NOR is composed of three superconducting flux qubits, which have all-to-all connectivity. The two types of NOR, consisting of the same superconducting circuits (*L* = 110 pH) with different *I*_c_, are prepared. In NOR2, heat treatment at 220 °C is applied after the fabrication to reduce *I*_c_ of the Josephson junction.

### JSIM analysis

The NOR circuit model is constructed and analyzed by a JSIM^27^. Owing to the time constraint, *T*_a_ is settled in 1 μs (see "Gray zone analysis in JSIM" in the Supplementary methods and Supplementary Fig. 2). The thermal noise current, which overcomes a trap to local minimum energy, is used. Each current condition is performed with 300 and 1000 iterations for the evaluation of the state diagram of NOR and the state transition in a single qubit, respectively. Details of the gray zone analysis are given in the “Gray zone analysis in JSIM” section of the Supplementary Methods.

## Supplementary Information

Supplementary Information.

## Data Availability

All data generated or analyzed during this study are included in this published article (and its supplementary information files).
