# Supplementary material for: Superconducting quantum circuit of NOR in quantum annealing
Source: Sci Rep. 2022 Sep 23;12:15894. doi: 10.1038/s41598-022-20172-0 (PMC9508137; doi:10.1038/s41598-022-20172-0)
Supplement: Supplementary file 1 — Supplementary Information. [file 41598_2022_20172_MOESM1_ESM.pdf]

Supplementary Materials for

Superconducting quantum circuit of NOR in quantum annealing

Daisuke Saida[1*], Mutsuo Hidaka[1], Kouhei Miyake[2], Kentaro Imafuku[1] and Yuki Yamanashi[2]

[1]National Institute of Advanced Industrial Science and Technology

[2]Yokohama National University

*Corresponding author: saida.daisuke@aist.go.jp

Supplementary Methods

Here, we discuss the process for deriving the theoretical degeneracy point. To give a general example of quantum annealing (QA), we consider the quantum dynamics governed by the following Hamiltonian

$$H = \sum_i h_i \sigma_z^{(i)} + \sum_{i>j} J_{ij} \sigma_z^{(i)} \sigma_z^{(j)} \ , \quad (1)$$

where $\sigma_z^{(i)}$ is z Pauli matrix acting on qubit i[1-3]. Energy can be evaluated using Eq. (1), where the qubit states of 1 and 0 are considered as values of +1 and -1, respectively. Based on this evaluation, we calculate the theoretical state diagram shown in Fig. 1(a), 1(d), and 1(g). In the NOR circuit with the problem Hamiltonian shown in Supplementary Fig. 1(a), the dimensionless local biases of h_i and the pairwise couplings J_{ij} take the following values

$$h_1 : h_2 : h_3 : J_{12} : J_{23} : J_{31} = \frac{1}{2} : \frac{1}{2} : 1 : \frac{1}{2} : 1 : 1. \quad (2)$$

Here, h_i and J_{ij} are given by

$$h_i = M_i \cdot I_{hi} \cdot I_{qi}$$
$$J_{ij} = M_{ij} \cdot I_{qi} \cdot I_{qj}, \quad (3)$$

where M_i is the mutual inductance between qubit i and the local bias line, I_{hi} is the current flowing through the local bias line, I_{qi} is the persistent current in qubit i, and M_{ij} is the mutual inductance between qubits i and j. By utilizing I_{h1}, each current can be written as

$$I_{h2} = \frac{h_2}{h_1} \cdot \frac{M_{31}}{M_{23}} \cdot \frac{J_{23}}{J_{31}} \cdot I_{h1}$$

$$I_{h3} = \frac{h_3}{h_1} \cdot \frac{M_1}{M_3} \cdot \frac{M_{23}}{M_{12}} \cdot \frac{J_{12}}{J_{23}} \cdot I_{h1}$$

$$I_{q1} = \frac{1}{h_1} \cdot \frac{M_1 M_{23}}{M_{12} M_{31}} \cdot \frac{J_{12} J_{31}}{J_{23}} \cdot I_{h1}$$

$$I_{q2} = \frac{1}{h_1} \cdot \frac{M_1}{M_{12}} \cdot J_{12} \cdot I_{h1}$$

$$I_{q3} = \frac{1}{h_1} \cdot \left(\frac{1}{M_{23}}\right)^2 \cdot \frac{{J_{23}}^2}{J_{31}} \cdot I_{h1}. \quad (4)$$

M_i takes the same value. Consequently, the current relationships at the degeneracy point of the NOR can be written as

$$I_{h2} = \frac{M_{31}}{M_{23}} \cdot I_{h1}$$

$$I_{h3} = \frac{M_1}{M_3} \cdot \frac{M_{23}}{M_{12}} \cdot I_{h1}. \tag{5}$$

Inductances L and mutual inductances M are extracted from the circuit layout utilizing InductEX[4] as summarized in Supplementary Table 1. We confirmed consistency both in L and M between the extracted values at InductEX and experiments.

In our method, the superconducting quantum circuit is composed of flux qubits with all-to-all connectivity by utilizing direct magnetic couplers. This configuration allows us to implement the Hamiltonian of NOR in the circuit directly without any redundant qubits. In contrast, a conventional quantum circuit like the chimera graph architecture needs five qubits to implement the Hamiltonian of NOR. In this implementation, redundant qubits are required to construct qubit interactions across unit cells. This brings a change in the annealing dynamics from the original problem Hamiltonian because the spectrum structure including the excited state is modified.

Experimental configuration. Each qubit state is evaluated by applying a time-dependent transverse field to the QA. The annealing effect is controlled by the annealing time (T_a) of the field. Arbitrary wave generators are used to apply flux with an accurate time schedule. To control the annealing effect, a transverse field is supplied through a current I_{trans} to the small loop (inserted in the main loop) in the qubit. The local biases of h_i in the Hamiltonian are controlled by applying the external bias current (I_h). To detect the qubit state, a flux-injection current (I_{QFP}) for a quantum flux parametron (QFP), a drive current (I_{bias}) for a dc superconducting quantum interference device (SQUID), and a modulation current (I_{flux}) for flux detection in the dc-SQUID are supplied to the readout. The QFP detects flux due to the persistent current in the qubit and transfers it to the dc-SQUID with amplification. The dc-SQUID is tuned by applying an external flux in response to the clockwise current in the qubit. The qubit state is evaluated over 10^4 iterations except for the measurement of the state diagram, where the qubit state is evaluated over 4×10^2 iterations in each current condition.

The superconducting quantum circuit is mounted in a dilution refrigerator and is cooled at 10 mK. We confirmed detection of the critical current (I_c) of 0.28 μA in the single Josephson junction, which is one order of magnitude smaller than that in NOR2, indicating that the electric noise is suppressed in our experiments.

The Type I gray zone is evaluated under current conditions equidistant from the degeneracy point. For example, the line profiles of L_1 and L_2 are analyzed in NOR2 with an (I_{h1}, I_{h2}) of (0-3.8, 3.0) and (3.0, 0-3.8) [μA], respectively. For the equidistant current step, 1.2 μA is considered. The Type II gray zone is analyzed along the diagonal direction. As shown in Fig. 1(c), (f), and (i), the position of the boundary generated along the diagonal direction changes with I_{h3}. The Type II gray zone between "001"-"110" is generated to the right along the diagonal direction and the width expands as I_{h3} increases without changing the position. On the other hand, the position of the Type II gray zone along the diagonal direction to the left is changed. We evaluate it where the boundary is generated.

Gray zone analysis in JSIM.

In the Josephson integrated circuit simulator (JSIM)[5] analysis, we should take care selection of a value of T_a from viewpoints of a time constraint and a risk of trapping in the local minimum energy. In order to overcome this trapping, we use a calculation model in which a thermal noise current flows through one of the Josephson junctions in the qubit. The thermal noise current is given by

$$\sqrt{\frac{4k_B T\Delta f}{R_S}},$$

where k_B is the Boltzmann constant, T is the temperature, Δf is a bandwidth, and R_s is the resistance. Supplementary Fig. 2 shows the thermal noise current dependence of the success probability of the JSIM analysis for $(A, B, R) = (0, 0, 1)$. The green circles, blue triangles and red squares correspond to the results analyzed with T_a of 1 μs, 10 μs and 100 μs, respectively. Here, iterations of each analysis in T_a of 1 μs, 10 μs and 100 μs are 1000, 1000 and 100 times, respectively. Without the thermal noise current, we cannot obtain appropriate results even if the analysis with T_a of 100 μs. The thermal noise current of approximately 1.9 pA/√Hz suits to escape the trapping in the local minimum energy in case of T_a of 100 μs. However, analysis with longer T_a (compared with T_a of 1 μs) requires significant time cost for the calculation. For a T_a of 1 μs, a thermal noise current of approximately 3.0 pA/√Hz is appropriate to overcome trapping in the local minimum state. Owing to the time constraint, T_a of 1 μs is used in the JSIM analysis.

In Fig. 3(d), the trends for an I_{h3} of 3-4 μA are different with a thermal noise current above 2.5 pA/√Hz or below 2.0 pA/√Hz. Here, data are overlapped between 1.5 pA/√Hz and 2.0 pA/√Hz. Data analyzed by 2.5 pA/√Hz and 3.0 pA/√Hz take slightly higher values. Degeneracy points are obtained around I_{h3} of 2.8 μA in cases of the noise current above 2.5 pA/√Hz. On the other hand, the degeneracy points are I_{h3} of 2.3 μA in cases of under 2.0 pA/√Hz. Under 2.0 pA/√Hz, trapping to the local minimum state occurs. Data for an I_{h3} of 2.5 μA with a thermal noise current of 2.5 pA/√Hz indicates a slight effect of the trapping from the local minimum state. We adopt the thermal noise current of 3.0 pA/√Hz for evaluation of the state diagram and the widths of the gray zones. Under I_{h3} of 2.3 μA, a slope in I_{h3} dependence of ΔI_{h1} is consistent among the noise current of 1.5-3.0 pA/√Hz. However, minimum values of ΔI_{h1} are varied. This is because amplitudes of the noise current affect to the gray zone, resulting in different minimum values. We define the required external bias current as the width of the gray zone until one of the logic components occupies a probability in the range between 0.1 and 0.9 in JSIM analysis. The equidistant current step of 1.2 μA, which is considered in the Type I gray zone evaluation from experiments, is adopted. As represented in Fig. 3, the minimum width of the gray zone is different between experiments and JSIM analysis. As described later, the impact of flux generated by surrounding circuits appears differently between JSIM and experiments, resulting in a difference in the minimum width of the gray zone. However, the consideration of the equidistant current step contributes to suppressing the effect of the generation of minor logic components.

Supplementary Note

Detailed characteristics of the NOR operation. Here, we present characteristics of the NOR1 (I_c = 6.25 μA). First, we investigate the external current (I_h) combinations by which the four candidate components are most frequently generated in experiments. Supplementary Fig. 3 shows the histogram for NOR1 at currents of $(I_{h1}, I_{h2}, I_{h3}) = (1.6, 1.6, 2.0)$ [μA], where all four components are observed with 10^4 iterations. As shown in Fig. 1(c), four logic regions of NOR are overlapped in this current condition. For the sake of convenience, we define this current condition as the experimental degeneracy point. This point works similar to the theoretical degeneracy point where the Hamiltonian takes four kinds of the lowest energy. In Supplementary Fig. 3, the annealing time (T_a) is modulated between 1 and 1000 μs. For a T_a of 100 μs, the distribution of logic components of NOR is close to even. In all experiments described later, a T_a of 100 μs is utilized. At the experimental degeneracy point, the logic components of NOR randomly occur, as shown in Supplementary Fig. 4. Note that we can obtain desirable logic components of NOR by applying an offset current α to this current condition. Biasing one of the qubits by adopting α in the initial condition restricts the state of the other qubit because the qubits interact with each other to minimize the energy after QA. Supplementary Figs. 5(a)–5(d) show histograms of the NOR component with a modulating α, which is set between 0.25 and 5.00 μA. Each logic component is obtained with a probability of success above 90%, indicating the possibility of a highly accurate operation based on our superconducting quantum circuit.

NAND operation. In the same circuit shown in Supplementary Fig. 1(b), the Hamiltonian takes minimum values in NAND

by adopting a negative value of the local bias current. Although the sign is inverted, the degeneracy point is similar to that in NOR. We would like to emphasize that basic logic components of NOR and NAND are achieved with the same superconducting quantum circuit by converting the sign of the local bias current. Supplementary Figs. 6(a)-6(c) show state diagrams in theory, JSIM analysis with I_{h3} = -2.0 μA, and experiments with I_{h3} = -2.0 μA at 10 mK, respectively. In this JSIM analysis, the thermal noise current is eliminated. The current conditions of the degeneracy point take the same values in experiments and JSIM analysis of (I_{h1}, I_{h2}, I_{h3}) = (-1.8, -1.8, -2.0) [μA]. In the state diagram, the boundary is generated between logic "101" and "011" and spreads along the diagonal in the left direction with an increase of I_{h3}. On the other hand, a boundary is generated between logic "001" and "110" and spreads from the degeneracy point along the diagonal in the right direction with a decrease of I_{h3}. These trends are reproduced in theory, JSIM analysis, and experiments. Desirable NAND components are produced by adopting the offset current to the degeneracy point (Supplementary Fig. 7). With an α of 1 μA, no error occurs in any logic element during the 10^4 iterations. A remarkable point in our method is the high accuracy, above 90%, in versatile logic components of NOR and NAND with the same circuit and without changing the central condition of the operation in absolute value.

Features of the Type I gray zone. Characteristics of the gray zone depend on the annealing time. Supplementary Fig. 9(a) and 9(b) show Type I gray zones for a T_a of 15 μs and 1 μs, respectively. As T_a becomes shorter, the the gray zone becomes wider. These features, namely, the existence of a wide boundary in L_1 and L_2 and a narrow boundary in L_3 and L_4, are the same regardless of the value of T_a. This suggests that the ease of changing the energy of logic components around the boundary is determined mainly by the external bias current I_h. On the other hand, we should take into account the intensity of the offset current when attempting to achieve an accurate operation because the width of the gray zone is modulated by T_a.

Calibration of the degeneracy point in experiment. NOR2 is the superconducting quantum circuit with an I_c of 3.75 μA obtained by adopting a thermal treatment of 220 degrees for NOR1. Owing to the reduction of the I_c, a dimensionless factor β_L becomes preferable for the qubit state control. Even in the largest injection of the flux during the QA, the energy potential does not have an inflection point with respect to flux. For this reason, it is expected that the experiment will be carried out under conditions closer to the theory. Supplementary Fig. 8 represents a histogram of logic components in a current condition of (I_{h1}, I_{h2}) = (1.6, 1.6) [μA] with a modulation of I_{h3} from 0 to 9 μA (current step is 0.2 μA). All candidate states in NOR are identified. The degeneracy point is estimated experimentally as (I_{h1}, I_{h2}, I_{h3}) = (1.6, 1.6, 2.8) [μA]. The state diagram in this current condition corresponds to Fig. 2. Supplementary Fig. 10(a) shows the state diagram analyzed by JSIM with a current condition of I_{h3} = 3 μA. Here, the thermal noise current of 3.0 pA/$\sqrt{}$Hz is considered. To evaluate the gray zone, the calculation is carried out 300 times in each current condition. Supplementary Fig. 10(b)-10(e) represent the line profiles of L_1, L_2, L_5, and L_6, respectively, which are depicted in Fig. 2(a). Owing to the thermal noise current, a transition area, which does not appear in Fig. 1(b), 1(e), and 1(h), is created around the boundary of each logic region. Though the effect is small enough for correct operation in NOR, an unintended logic component of (A, B, R) = (1, 1, 1), which is not the minimum state in the Hamiltonian, is found. The most important point is that by adopting the thermal noise current, the degeneracy point matches between theory and JSIM analysis. This indicates that by implementing the Hamiltonian to the circuit natively and correctly, accurate control of the superconducting quantum circuit is possible along with prediction of the operating point. On the other hand, we should take care in the analysis of the experimental degeneracy point. The difference between JSIM analysis and experiments is how the contribution of the flux generated by surrounding circuits is treated. Supplementary Fig. 11 shows the state-1 probability, corresponding to the state transition from 1 to 0, of each qubit composing the superconducting quantum circuit in NOR. As represented in Supplementary Fig. 1(b), each qubit is coupled with a readout circuit. During QA, the currents I_{trans} and I_h for a transverse field and the external bias current are supplied to the circuit for the qubit. Currents

I_{QFP}, I_{flux}, and I_{bias} are also supplied to the circuit of the readout for a flux injection to the QFP, flux modulation, and bias of the dc-SQUID, respectively. It should be noted that the flux induced by I_{trans}, I_h, I_{QFP}, I_{flux}, and I_{bias} affect each qubit because qubits and their associated readouts are statically coupled. This results in a difference of an offset flux in cases where the state-1 probability of each qubit is either solely biased or fully biased (the latter case corresponds to the flux condition at the NOR operation). Supplementary Fig. 11(a) shows the state-1 probability of NOR1. Supplementary Fig. 11(b) and 11(c) show the state-1 probability in each qubit of NOR2 in cases where it is solely and fully biased to the surrounding circuit, respectively. In the case of it being fully biased to the surrounding circuit, the state-1 probability of each qubit is distributed with respect to the external bias current due to the impact of the offset flux H_{sur}. Note that this offset current does not appear in JSIM analysis (Supplementary Fig. 11(d)). The gray zone of the state-1 probability, which corresponds to the width of I_h for a complete state transition, varies due to the flux from the surrounding circuit in JSIM analysis. The calibration of the impact of the offset flux, which affects the distribution of the state-1 probability, is required in experiments. Based on the state-1 probability of qubit-1 (A) and the relative offset in qubit-2 (B) and qubit-3 (R), calibrations of the experimental degeneracy point are carried out, resulting in (I_{h1}, I_{h2}, I_{h3}) = (1.6, 1.7, 3.0) [μA] at NOR1 and (I_{h1}, I_{h2}, I_{h3}) = (1.6, 1.6, 2.9) [μA] at NOR2. The calibrated degeneracy point is close to that of theory and JSIM analysis, indicating a native and correct implementation of the Hamiltonian to the superconducting quantum circuit.

Suppression of electric noise toward true quantum annealing. In order to achieve true QA, the suppression of noise effects, including thermal noise, magnetic flux noise from the environment, and electric noise, is vital. The thermal energy at 10 mK is estimated to be 1.4×10^{-25} J. The energy in the potential of the rf-SQUID in the qubit around its bottom after QA is about 2.0×10^{-21} J. The disturbance due to the thermal energy is negligible in our experiment. To suppress flux trapping due to the magnetic flux noise, we use a magnetic shield that surrounds the circuit. The state transition of the qubit shown in Supplementary Fig. 11 proves the elimination of flux trapping in experiment. In order to suppress the electric noise from instruments, a low-noise dc power supply with a noise floor of order fA/$\sqrt{\text{Hz}}$ is used. The external bias current I_h is supplied to the circuit of the qubit using this instrument, which has a resistance of 1 MΩ and behaves as a constant-current source. In this case, the energy of the electric noise is estimated to be around the order of 10^{-28} J, which is negligibly small compared with the thermal energy at 10 mK. Consequently, the annealing time dependence clearly appears in the state-1 probability of the qubit shown in Supplementary Fig. 11(e). The width of the gray zone is reduced around 0.4 μA in Type I for the case of an evaluation carried out with a current step of 0.2 μA. On the other hand, we cannot distinguish the annealing time dependence in the state-1 probability of the qubit when a dc power supply with a noise floor of order pA/$\sqrt{\text{Hz}}$ is used (Supplementary Fig. 11(f)). In this case, the energy of the electric noise is estimated to be around the order of 10^{-22} J, which is close to the energy in the rf-SQUID. This results in an expanding of the gray zone in the state-1 probability. Supplementary Fig. 12 shows the state diagram obtained by this instrument. The Type I gray zone expands widely up to 1 μA, which is close to the width evaluated in experiment with a dc power supply with a noise floor of order fA/$\sqrt{\text{Hz}}$ carried out at 4.2 K. This means that a large offset current above 1 μA is required to correctly obtain each logic component. However, the quantum annealing effect is buried under the electric noise. The suppression of electric noise is important for achieving high accuracy and expandability of circuit scaling in QA. Thus, the instrument selection for I_h is crucial. Using the current source with a noise floor on the order of fA/$\sqrt{\text{Hz}}$ for I_h, the transition rate of the state-1 probability depends on T_a and is accelerated with longer T_a during the interval from 20 μs to 1 ms in our setup. This acceleration is probably related to quantum tunneling. The experimental features of superconducting flux qubits agreed well with the results of quantum mechanical simulations in a D-wave system with fewer than 8 qubits[6]. Because the energy scale of our qubit is similar to that used in Ref. 6, the quantum effect, shown in the simulation performed using D-wave systems, also appears in our experiment because of the noise suppression.

Expandability of the circuit. An arbitrary Hamiltonian can be described based on the circuit SAT expression[7,8]. The ground-state spin logic[9] allows us to obtain the Hamiltonian when the input and output relationships are expressed by Boolean logic gates (cf : NOR, NAND, AND and OR[10]). NOR and NAND are versatile logic elements, and these are implementable with high accuracy using the superconducting quantum circuit in QA. In order to expand the circuit, each logic element is combined with a connection qubit. Supplementary Fig. 13 shows the evaluation of an error rate in the case of a three qubit system, where qubits Q_a and Q_b are combined via the connection qubit. Q_a and Q_b correspond to the output of the first NOR element and one of the inputs of the second NOR element, respectively. The error rate depends on the inductance L_{hc} of the connection qubit. JSIM analysis is carried out with 100 iterations at each L_{hc}. A smaller L_{hc}, but slightly larger than that in the NOR qubit, brings no error for the connection. This means that a superconducting quantum circuit having a wide range of applications will be possible with high expandability in QA.

L1	109.7 pH
L2	109.9 pH
L3	111.0 pH
M1	31.8 pH
M2	31.8 pH
M3	31.8 pH
M12	-10.3 pH
M23	-22.0 pH
M31	-22.0 pH

Supplementary Table 1 | Extracted values of *L* and *M* in the superconducting quantum circuit of NOR.

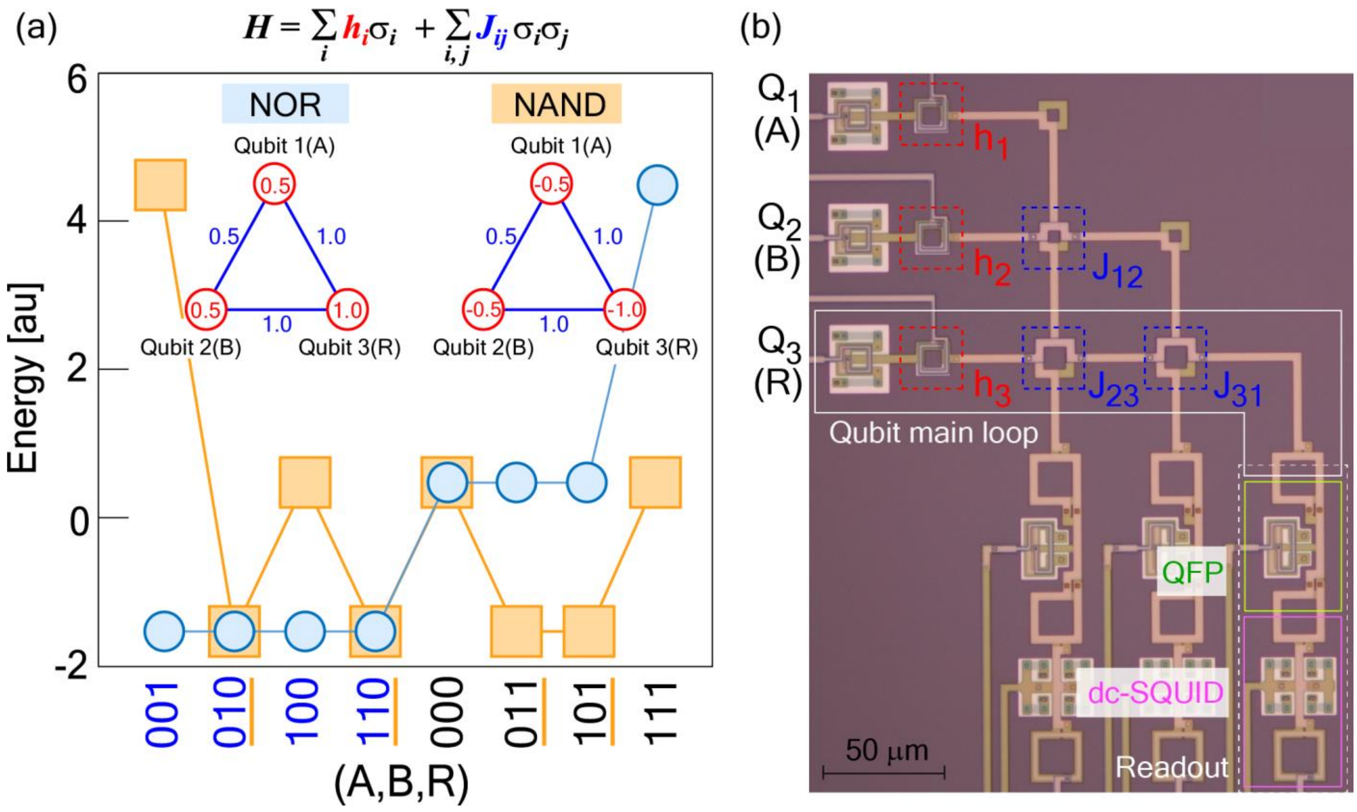

Supplementary Figure 1 | (a) Energy of each state in the NOR and NAND. J_{ij} terms of the Hamiltonian are implemented by tuning the overlapping area between qubits *i* and *j* in Fig. (b). After quantum annealing, each qubit state takes combinations to reach the Hamiltonian minimum energy. **(b)** The superconducting quantum circuits for NOR (NAND). The NOR (NAND) consists of three superconducting flux qubits (Q_i), which have all-to-all connectivity. The qubit state is detected by a readout circuit composed of a quantum flux parametron (QFP) and a dc superconducting quantum interference device (SQUID).

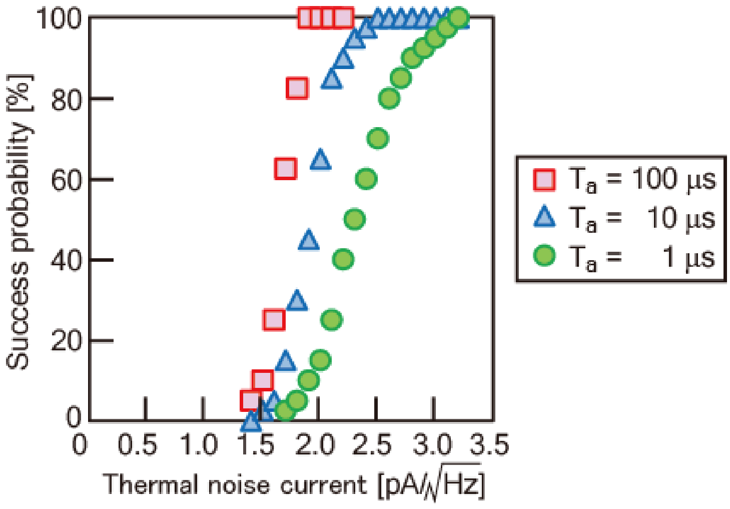

Supplementary Figure 2 | Thermal noise current dependence of success probability in a case of (*A*, *B*, *R*) = (0, 0, 1) at the JSIM analysis.

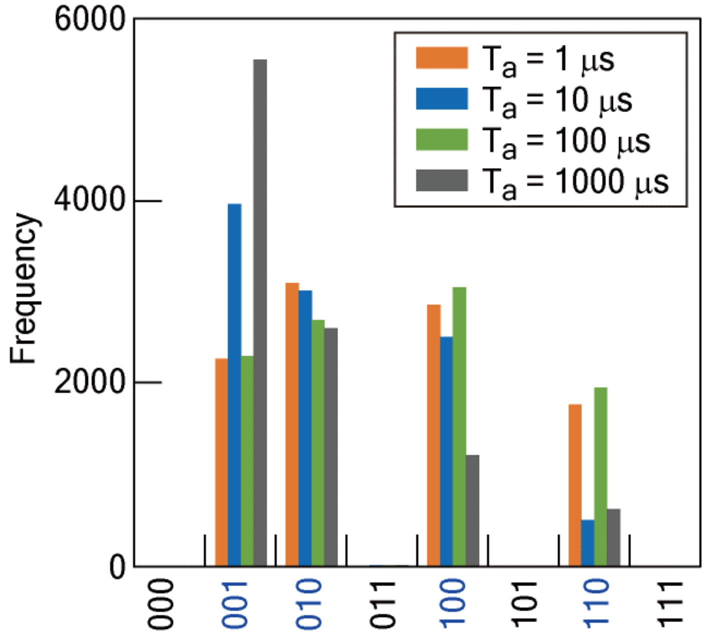

Supplementary Figure 3 | Frequency distribution of each logic component in the experiments at 10^4 iterations using NOR1 (I_c = 6.25 μA). T_a is modulated between 1 and 1000 μs. All four components are observed in every experiment.

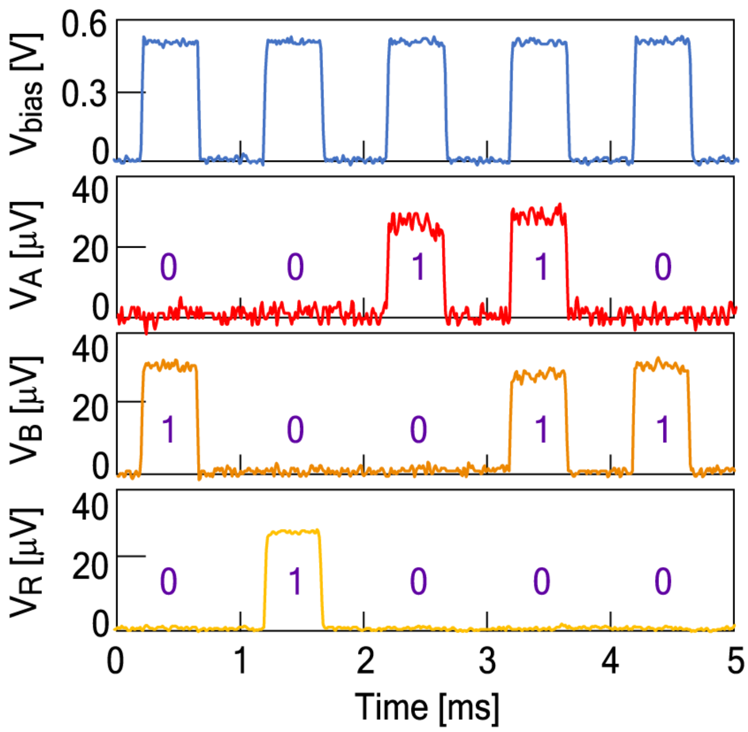

Supplementary Figure 4 | Experimental demonstration of the random operation of NOR at the degeneracy point using NOR1. The logic components of NOR generated randomly synchronized with the trigger signal (V_{bias} is bias for the dc-SQUID).

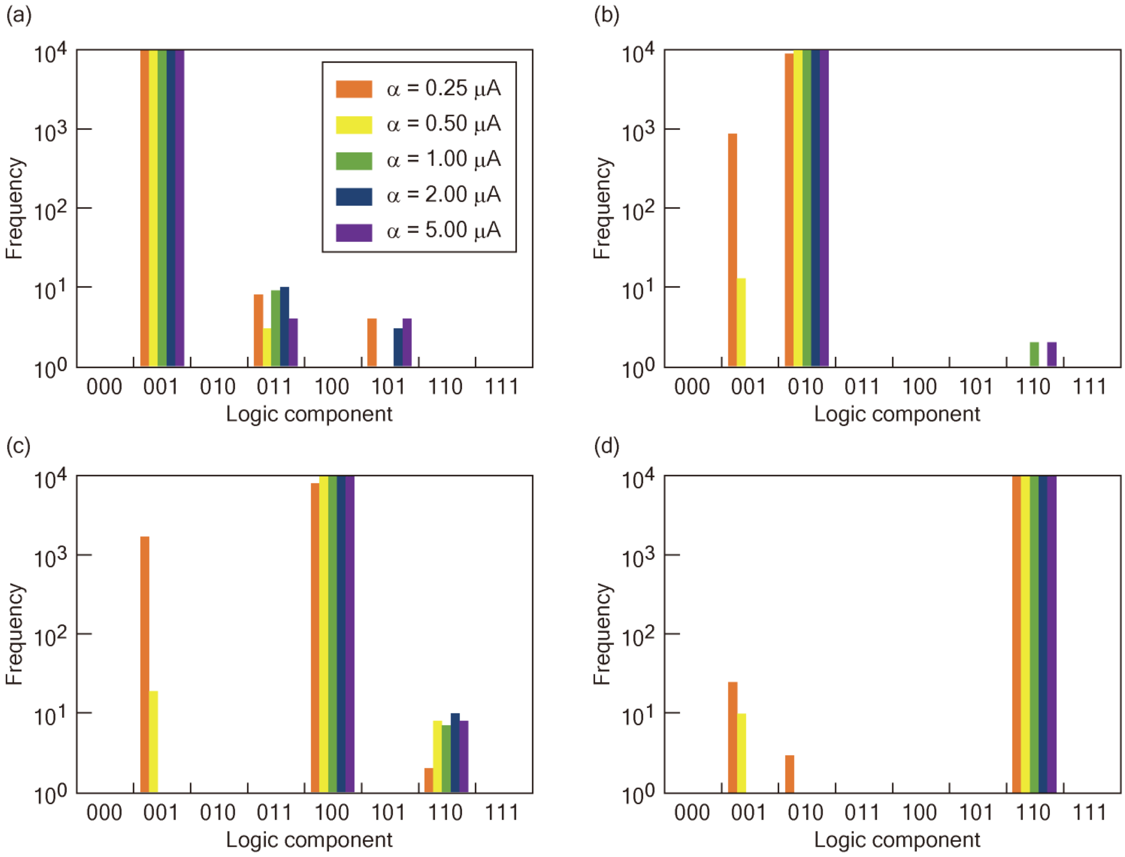

Supplementary Figure 5 | Experimental demonstration of the NOR with an (*A*, *B*, *R*) of **(a)** (0,0,1), **(b)** (0,1,0), **(c)** (1,0,0), and **(d)** (1,1,0). NOR1 is utilized.

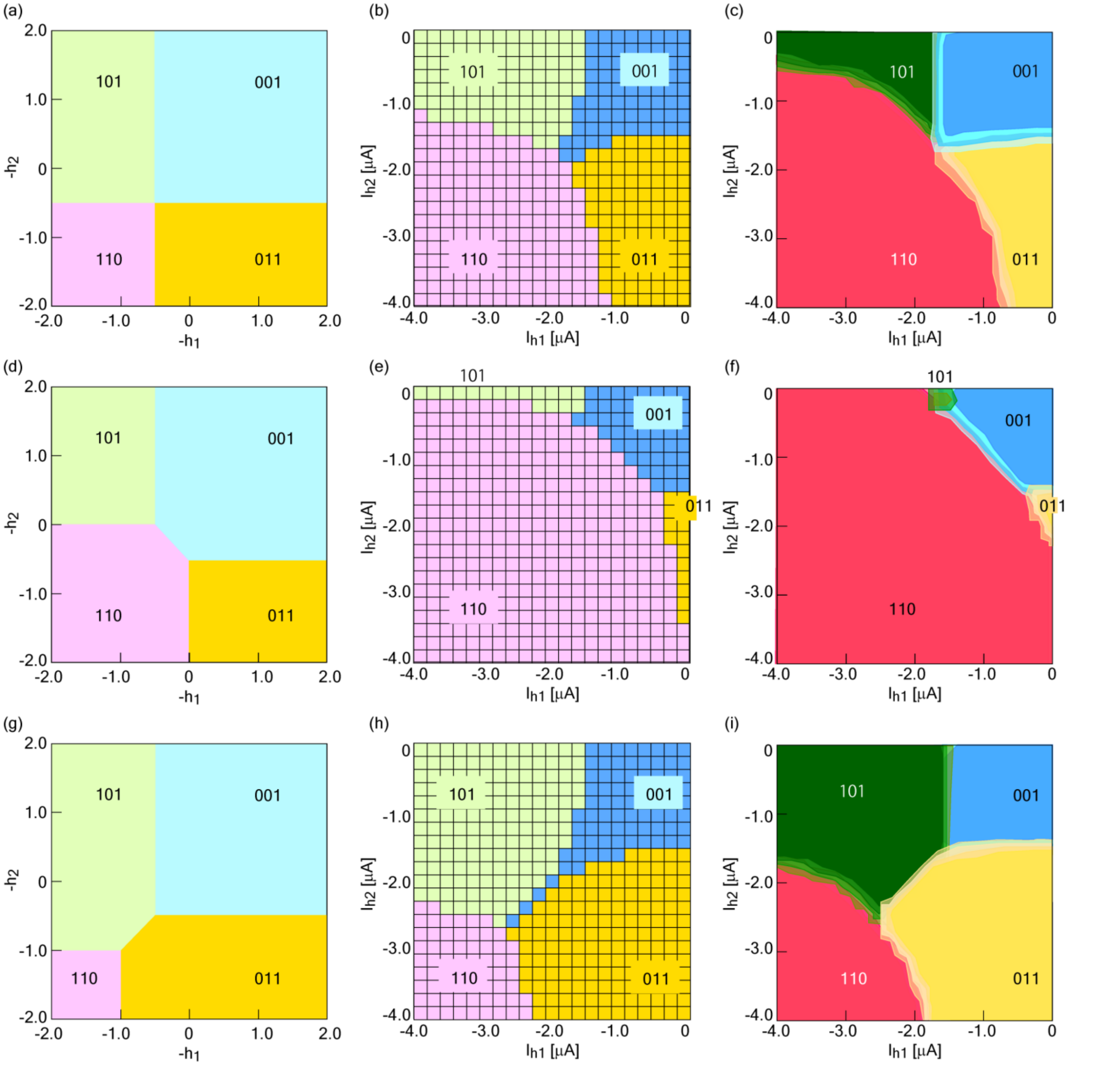

Supplementary Figure 6 | State diagram of NAND operation. **(a)**, **(b)**, and **(c)** correspond to the state diagram in theory, JSIM analysis with thermal noise current, and the result in the 10 mK experiment, respectively, with I_{h3} = -2 μA. **(d)**, **(e)**, and **(f)** correspond to the state diagram in theory, JSIM analysis with thermal noise current, and the result in the 10 mK experiment, respectively, with I_{h3} = -1 μA (the absolute value is reduced 50% from the degeneracy condition). **(g)**, **(h)**, and **(i)** correspond to the state diagram in theory, JSIM analysis with thermal noise current, and the result in the 10 mK experiment, respectively, with I_{h3} = -3 μA (the absolute value is increased 50% from the degeneracy condition). NOR1 is utilized.

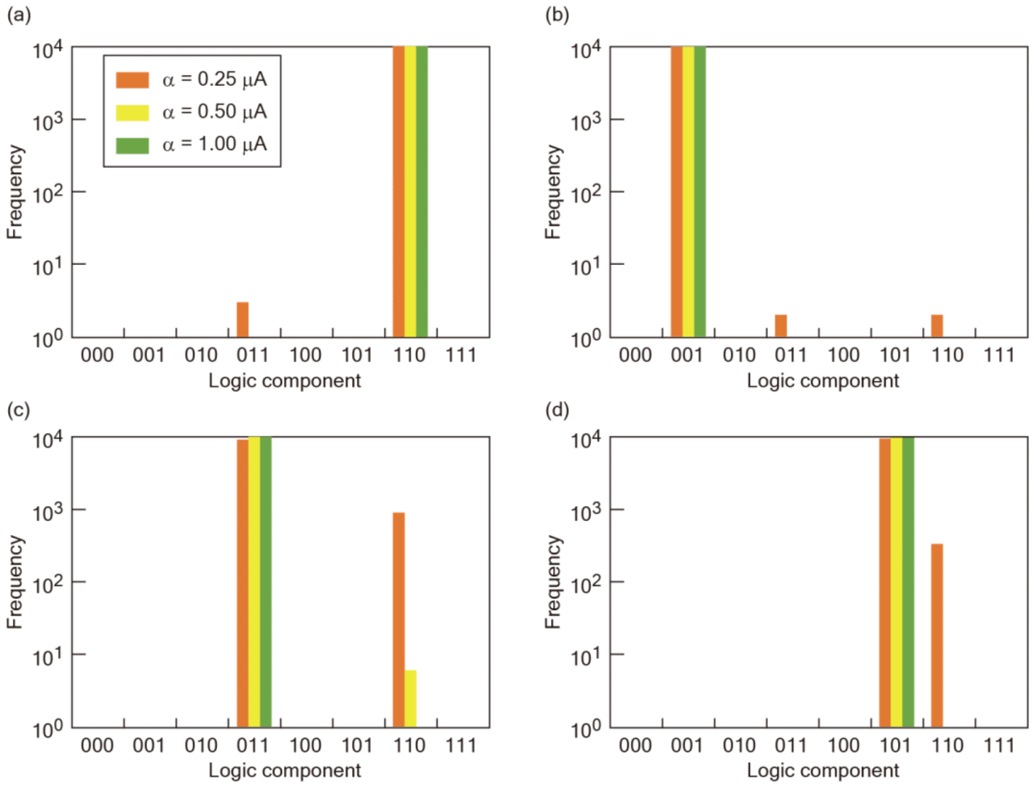

Supplementary Figure 7 | Experimental demonstration of the NAND with an (A, B, R) of **(a)** (1,1,0), **(b)** (0,0,1), **(c)** (0,1,1), and **(d)** (1,0,1). NOR1 is utilized.

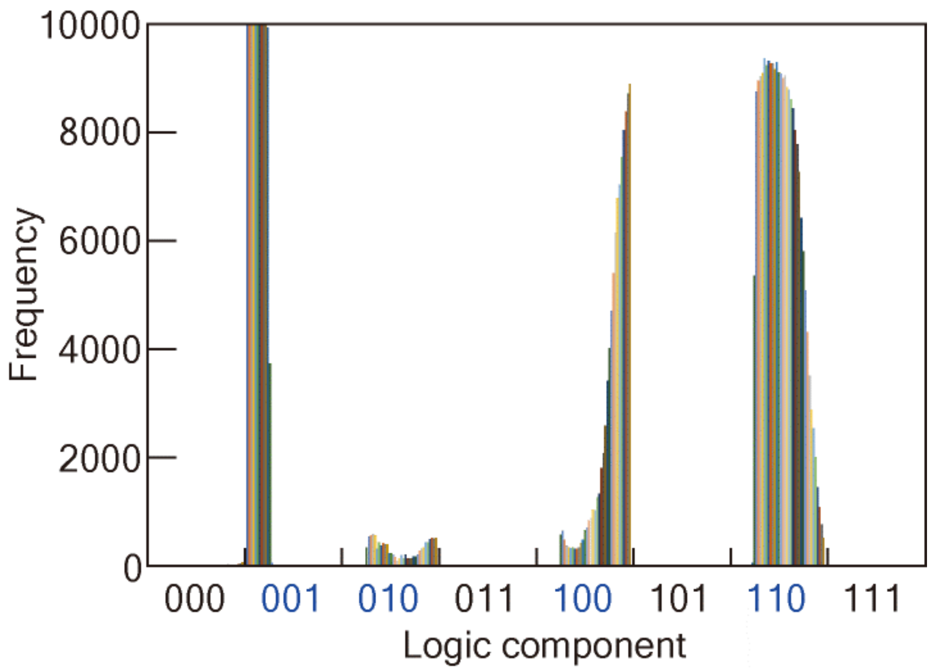

Supplementary Figure 8 | Frequency distribution of each logic component in the experiments at 10^4 iterations using NOR2 (I_c = 3.75 μA) with a T_a of 100 μs and a current condition of (I_{h1}, I_{h2}) = (1.6, 1.6) [μA]. Here, the value of I_{h3} is modulated from 0 to 9 μA with a 0.2 μA step. All four components are observed in both experiments. We evaluate the degeneracy point as (I_{h1}, I_{h2}, I_{h3}) = (1.6, 1.6, 2.8±0.2) [μA].

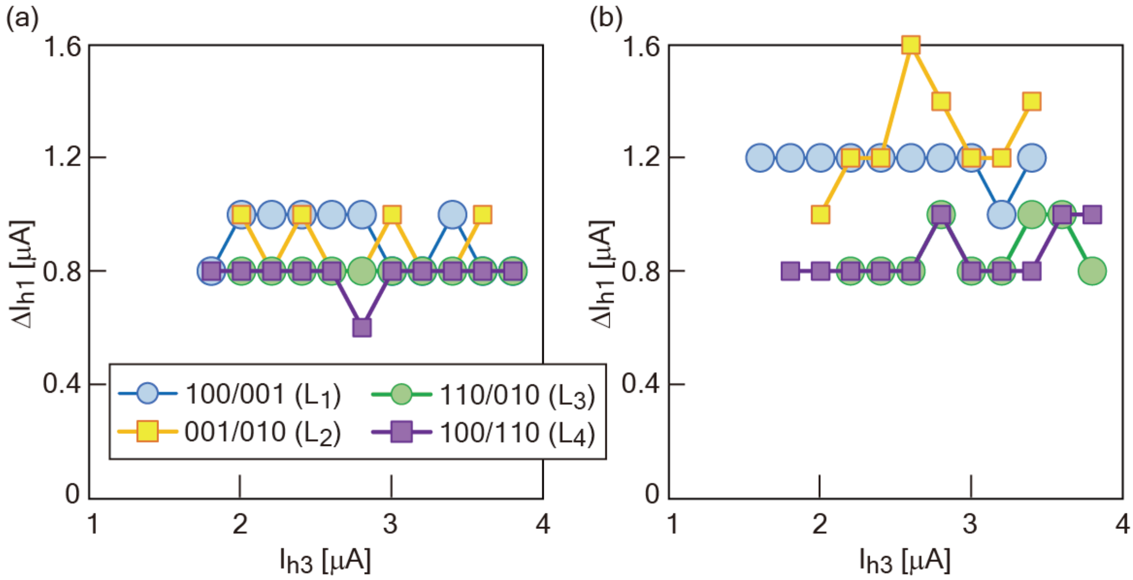

Supplementary Figure 9 | Type I gray zone analysis with (a) T_a = 15 μs and (b) 1 μs in experiment at 10 mK. The gray zone of T_a = 100 μs is shown in Fig. 3(c). The width of the gray zone depends on the annealing time, indicating that the impact of quantum tunneling, which contributes to converging the energy, is minimized.

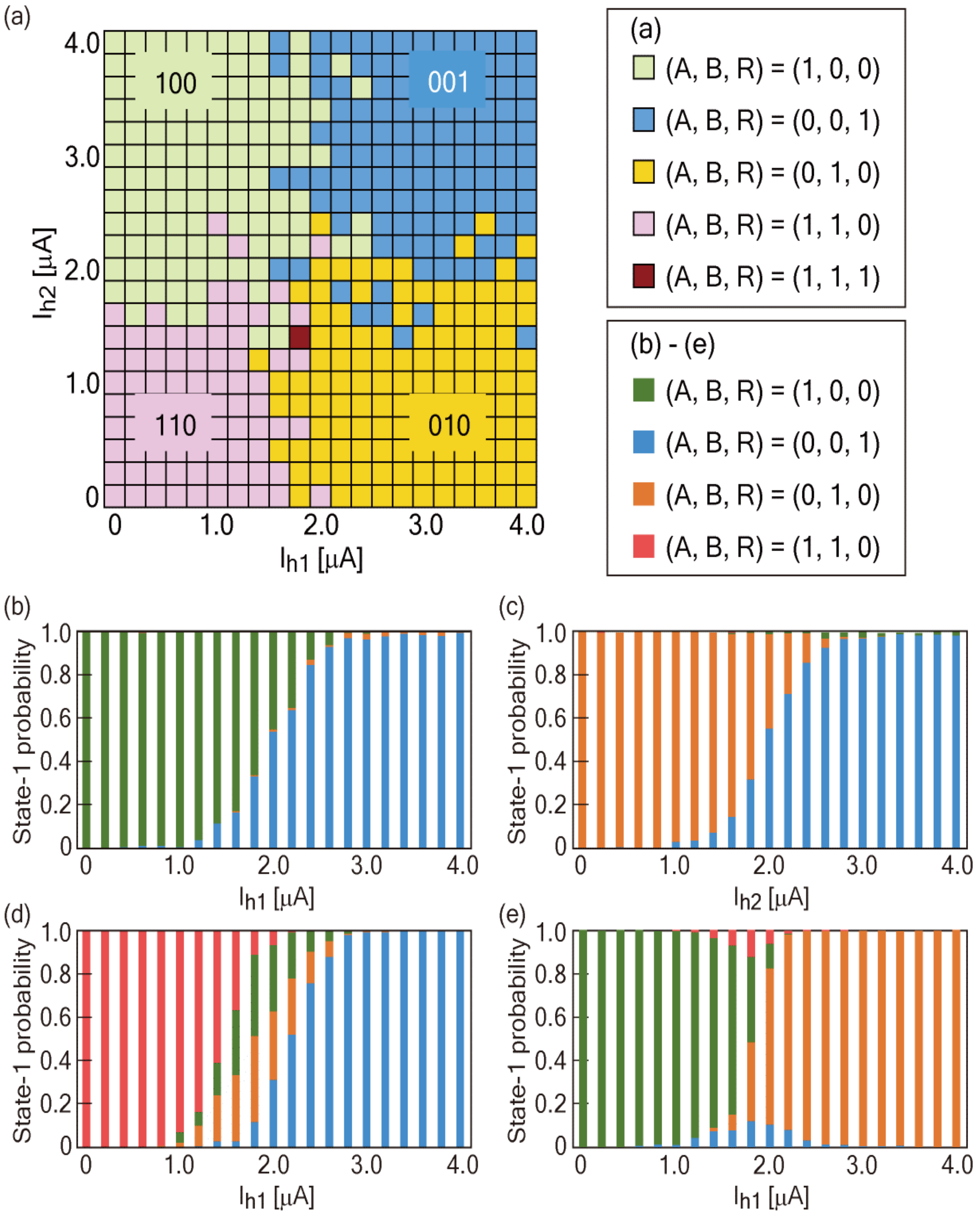

Supplementary Figure 10 | (a) State diagram of NOR at I_{h3} = 3.0 μA in JSIM analysis. Here, the thermal noise current of 3.0 pA/$\sqrt{\text{Hz}}$ is considered. The frequency distribution at the line profile described in Fig. 2(a) of **(b)** L_1, **(c)** L_2, **(d)** L_5, and **(e)** L_6. L_1 is a line profile along I_{h2} = 2.8 μA. L_2 is a line profile along I_{h1} = 2.8 μA. L_5 and L_6 are line profiles observed along the diagonal direction to the right and left, respectively.

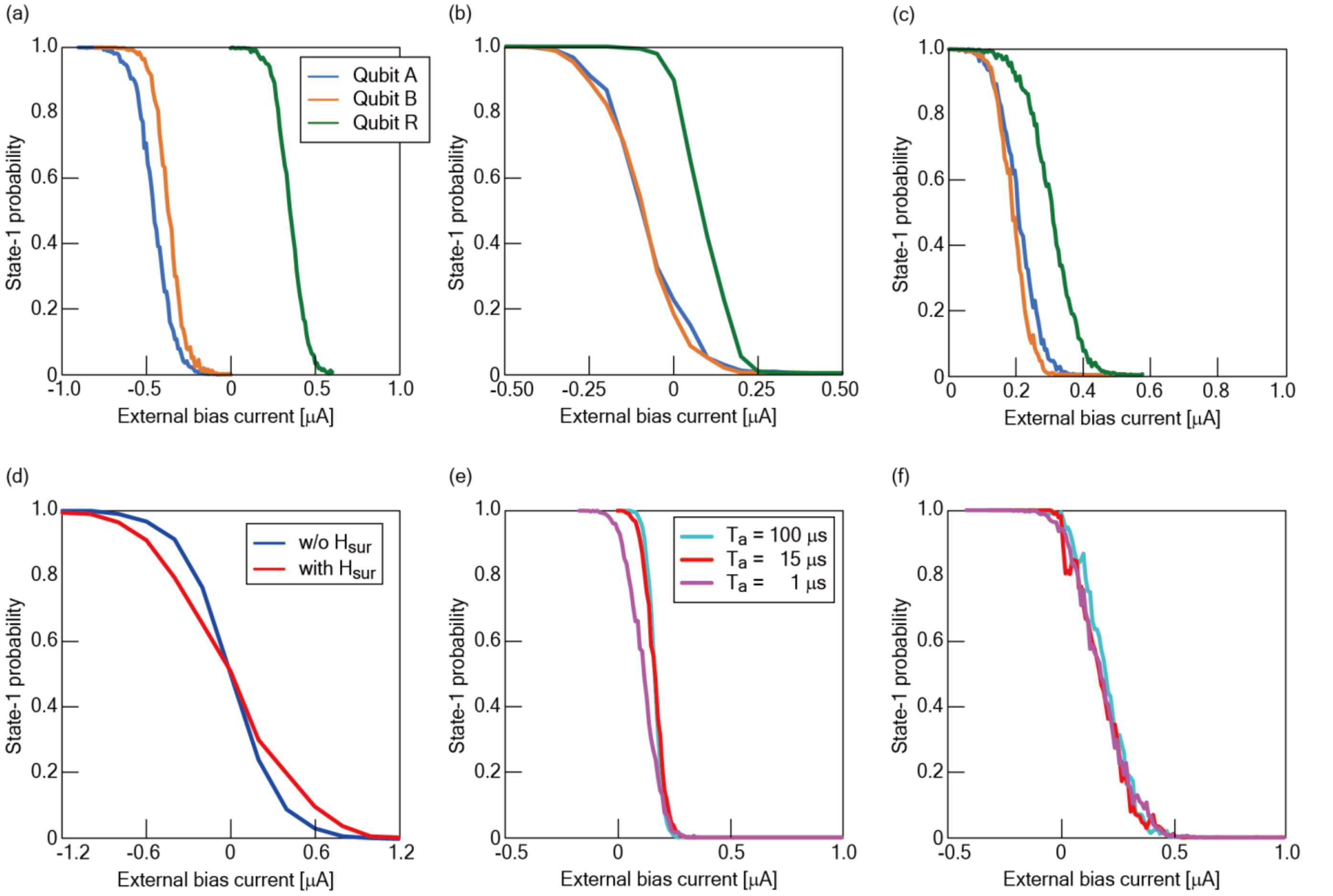

Supplementary Figure 11 | (a) State-1 probabilities of each qubit in NOR1. In order to estimate the impact of the offset flux in the measurement of a NOR operation in the superconducting quantum circuit, the surrounding circuits are fully biased during evaluation of the state transition in each qubit. **(b)** State-1 probabilities of each qubit in NOR2. The qubit and its coupled readout circuit is solely biased during the evaluation of the state transition. **(c)** State-1 probabilities of each qubit in NOR2. All qubits and coupled readout circuits are fully biased during evaluation of the state transition. **(d)** State-1 probabilities calculated by JSIM with and without the flux generated by surrounding circuits (H_{sur}). **(e)** Annealing time dependence of the state-1 probabilities utilizing a current source with a noise floor of fA/$\sqrt{\text{Hz}}$. **(f)** Annealing time dependence of the state-1 probabilities utilizing a current source with a noise floor of pA/$\sqrt{\text{Hz}}$.

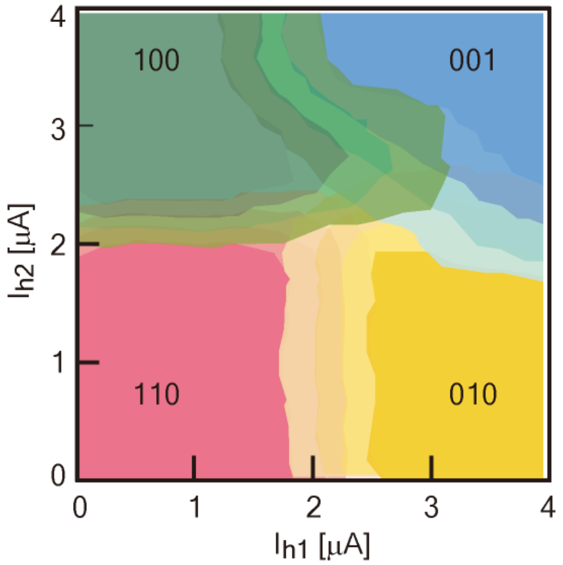

Supplementary Figure 12 | State diagram of the NOR operation. I_{h1} and I_{h2} are supplied using a current source with a noise floor of pA/$\sqrt{}$Hz.

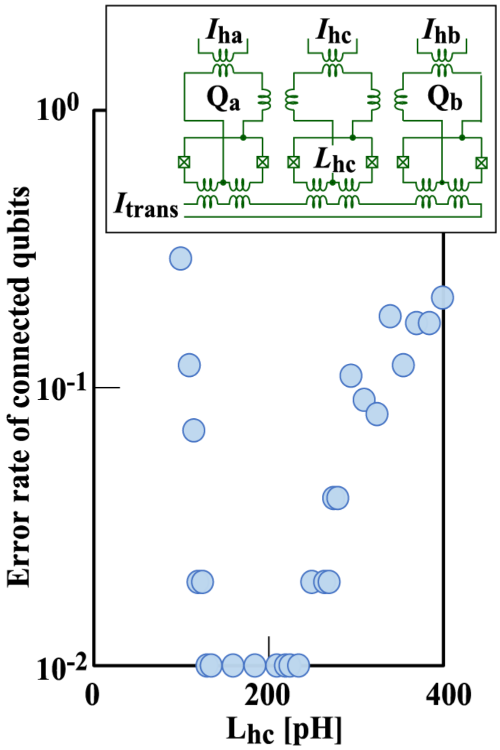

Supplementary Figure 13 | Error rate evaluation of a three qubit system between Q_a and Q_b via a connection qubit in JSIM analysis.

Supplementary References

1. Johnson, M., *et al*. Quantum annealing with manufactured spins. *Nature* **473**, 194-198 (2011).

2. Harris, R., *et al*. Synchronization of multiple coupled rf-SQUID flux qubits. *New J. Phys*., **11**, 123022 (2009).

3. Harris, R.. *et al*. Compound Josephson-junction coupler for flux qubits with minimal crosstalk. *Phys. Rev. B* **80**, 052506 (2009).

4. Fourie, C., Wetzstein, O., Ortlepp T. & Kunert, J. Three-dimensional multi-terminal superconductive integrated circuit inductance extraction. *Supercond. Sci. Tech*., **24**, 125015 (2011).

5. Fang, E. S. & Van Duzer, T., A Josephson integrated circuit simulator (JSIM) for superconductive electronics application. in *Proc. Ext. Abstr. 2nd Int. Supercond. Electron. Conf.* 407 (1989).

6. Boixo, S., *et al*. Computational multiqubit tunnelling in programmable quantum annealers. *Nat. Commun.* **7**, 10327-10334 (2016).

7. Pudenz, K., Tallant, G., Belote, T. & Adachi, S. Quantum annealing and the satisfiability problem. Preprint at https://arxiv.org/pdf/1612.07258.pdf (2016).

8. Babbush, R., Perdomo-Ortiz, A., O'Gorman, B., Macready, W. & Aspuru-Guzik, A. Construction of energy functions for lattice heteropolymer models: efficient encodings for constraint satisfaction programming and quantum annealing. https://arxiv.org/abs/1211.3422 (2012).

9. Whitfield J. Faccin M. and Biamonte J. Ground-state spin logic, *EPL*, **99** 57004 (2012).

10. Saida, D., et al. Experimental demonstrations of native implementation of Boolean logic Hamiltonian in a superconducting quantum annealer. *IEEE Trans. Quant. Eng*. **2**, 3103508-3103515 (2021).
